# Risk stratification in PAH

**DOI:** 10.21542/gcsp.2020.9

**Published:** 2020-04-30

**Authors:** Paul A. Corris

**Affiliations:** Emeritus Professor of Thoracic Medicine, Newcastle University, Newcastle, UK

## Introduction

Pulmonary arterial hypertension (PAH) is a chronic disease of the pulmonary vasculature characterized by progressive narrowing of the pulmonary arteries leading to increased pulmonary vascular resistance, right heart failure, and ultimately premature death^1^.

There has been a significant improvement in the available medical therapeutic options in this field that have impacted the short-term survival and morbidity in these patients^2^. However, the median survival post-diagnosis remains unacceptable at 7 years^3^.

Physicians’ ability to predict PAH disease progression and risk allows them to determine the patient’s prognosis, make informed adjustments to therapy, and monitor his or her response to therapy^4^. If widely adopted, risk prediction can enhance the consistency of treatment approaches and improve the timeliness of referral for lung transplantation. This approach should lead optimal, directed care that ultimately reduces morbidity and improves mortality in patients with PAH.

## Important factors for risk stratification

Risk stratification should have a multifaceted approach that includes both objective and subjective variables that ultimately create a profile for an individual patient that is able to provide accurate prognostic information. Such a profile will then provide the basis for appropriate adjustments in therapy leading to improved outcomes. The individual variables should be statistically validated and evidence based. The following variables have all been demonstrated to have implications for patient outcomes:

### Demographics

Within PAH, there are certain subtypes of patients that have a worse prognosis. These include age (>60 years), male gender, systemic connective tissue disease, and the bone morphogenetic protein receptor II mutation^5–8^.

### Functional class and capacity

Functional class is an easily accessible risk variable that can be obtained at every clinic visit. This self-reporting system of symptoms is a subjective measure made by the examining physician and has been criticised for this reason. However it has stood out as a consistent and effective clinical tool, representing the continuum of disease and able to discriminate prognosis effectively. Patients who have a lower functional class according to WHO criteria (I or II) at baseline have a better prognosis than those who are functional class III or IV. Changes in 6-minute walk distance have not been shown to predict survival, although an improvement or deterioration in functional capacity plays an important role in contributing to decisions to initiate, maintain, or escalate therapy. A threshold of 440 m is suggestive of a distinction between high-and low-risk patients in pulmonary hypertension guidelines^6,8^. Reduced exercise capacity noted on exercise testing also indicates a worse prognosis. Syncope, considered a marker of class IV symptoms, carries adverse prognostic relevance in PAH.

### Laboratory testing

Plasma brain natriuretic peptide (BNP) is secreted by the left and right ventricles when the cardiac muscle is under stress and has been demonstrated to provide an independent predictor of mortality in patients with PAH^9^.

The degree of right ventricular dysfunction in patients with PAH correlates with increasing levels of BNP. Recent evidence supports an optimal BNP threshold of 340 pg/mL strongly predicts 5-year survival in patients with PAH (hazard ratio 3.6; 95% confidence interval, 3.0–4.2; p < 0.001)^10^. Additionally, elevated levels of creatinine, total bilirubin, uric acid, and troponin, along with decreased albumin and serum sodium, are all markers of worse outcomes in patients with PAH^4^.

### Imaging

An echocardiogram is a vital imaging tool in screening for pulmonary hypertension and assessing the right ventricular size and function in patients with PAH. A tricuspid annular plane systolic excursion of <1.8 cm, right atrial size >18 cm^2^, and the presence of pericardial effusion are all known to suggest high-risk patients^1^. There is ever-increasing evidence supporting cardiac magnetic resonance imaging as a very useful tool to monitor a patient’s prognosis and risk. In particular, assessment of right ventricular size (specifically end diastolic volume morphology) and global estimates of function have a good evidence base.

### Hemodynamics

A right heart catheterization is vital for accurate diagnosis in PAH as well as providing prognostic information. Known prognostic parameters include high right atrial pressure (>14 mmHg), pulmonary vascular resistance (>5 WU), venous oxygen saturation <60%, and low cardiac index (<2 L/min/m^2^)^8^.

### Hospitalizations

All-cause hospitalization, especially related to PAH events, within 6 months is associated with an increased risk of mortality and recurrent hospitalizations^10^.

**Table 1:** The REVEAL RISK SCORE calculators. A is the original REVEAL score and C the updated REVEAL 2 score.

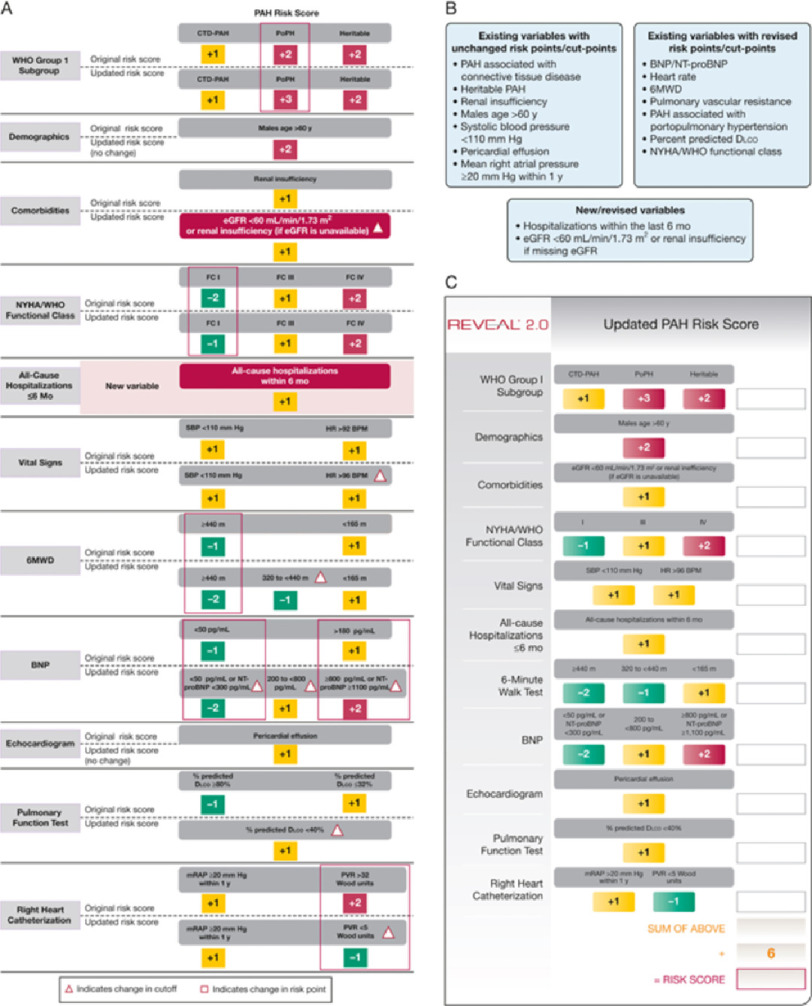

**Figure 1.**
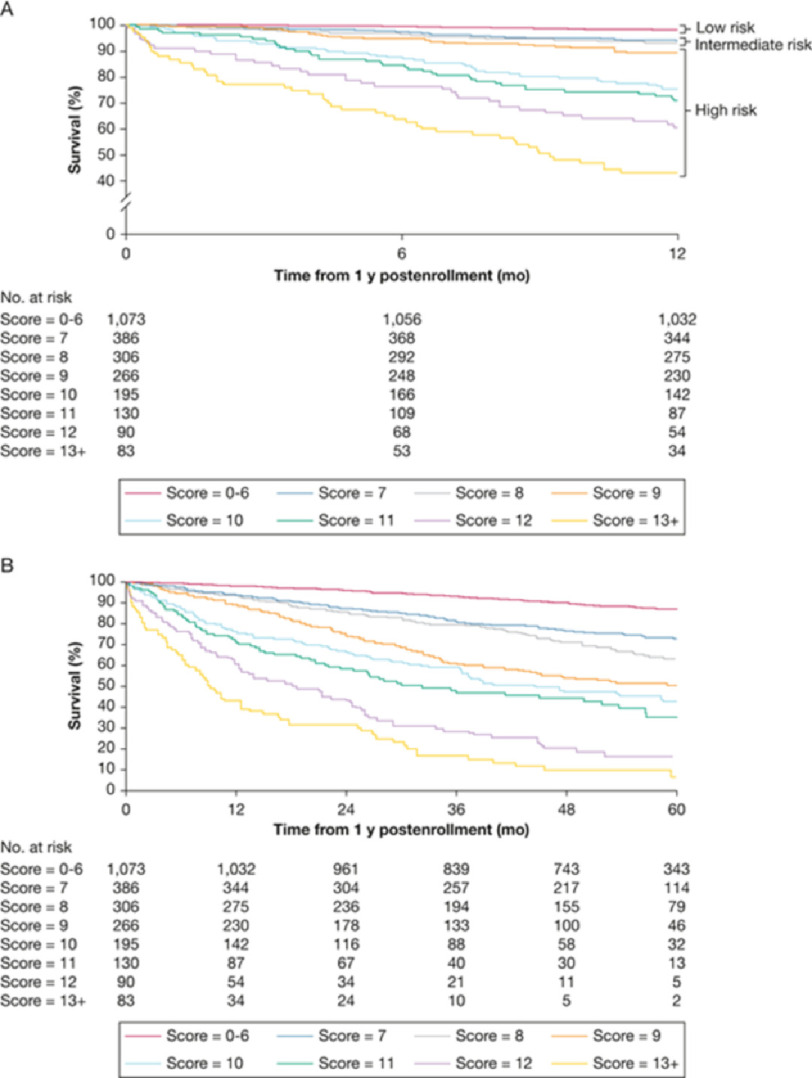
Use of REVEAL (A) and REVEAL 2(B) scores in determining prognosis.

## Tools for risk stratification

There are various risk calculators that are available to risk stratify patients with PAH that all focus on different aspects of the disease process. The primary aim of these assessments is to project patient trajectory based on available information, allowing for informed and individualized decision-making. Ideally, these tools should be multifaceted, applicable along the continuum of disease, easy to use, and validated. Analysis of the Registry to Evaluate Early and Long-term PAH Disease Management (REVEAL) data produced a versatile risk calculator based on over 2,500 PAH registry patients who were newly and previously diagnosed with PAH (Table 1) (Figure 1)^6,10^.

Several of the European PAH registries (French Pulmonary Arterial Hypertension Network registry, Spanish Registry Of Pulmonary Arterial Hypertension, Swedish Pulmonary Arterial Hypertension Registry, and Comparative, Prospective Registry of Newly Initiated Therapies for Pulmonary Hypertension) have developed algorithms calculating risk. These aim to stratify patients as low, intermediate, or high risk of death and are represented in the 2015 European Society of Cardiology and European Respiratory Society pulmonary hypertension guidelines (Table 2)^8,11,12^.

**Table 2:** ESC/ERS risk score.

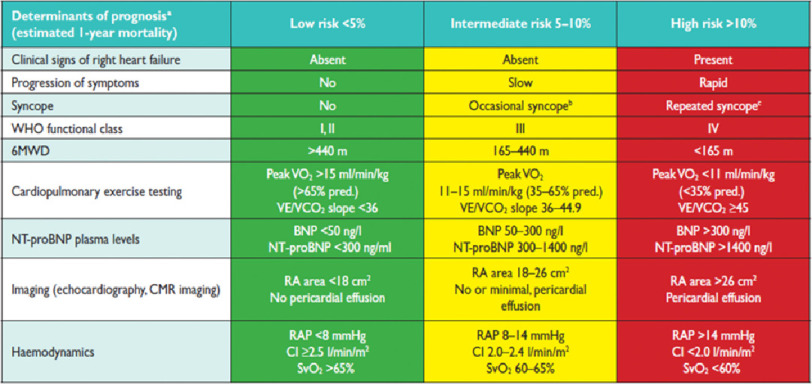

**Figure 2.**
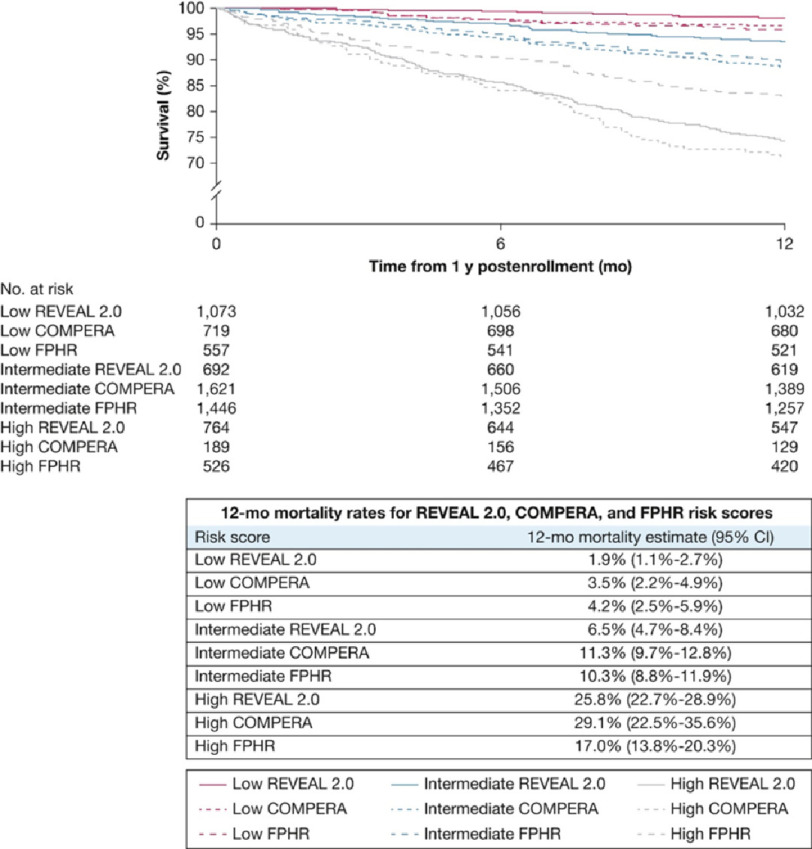
Comparison of survival estimates using REVEAL, COMPERA, and French methods of evaluating risk.

There have been two approaches to calculating an overall score, both of which have been validated. The French group and COMPERA registry group counted the number of low risk measures using three variables namely functional class, 6 minute walking distance and NT pro-BNP level. The Swedish group proposed an average score ascribing 1 for low, 2 for intermediate, and 3 for high risk (a low risk variable scores 1, intermediate 2, and high 3) (Figure 2).

**Figure 3.**
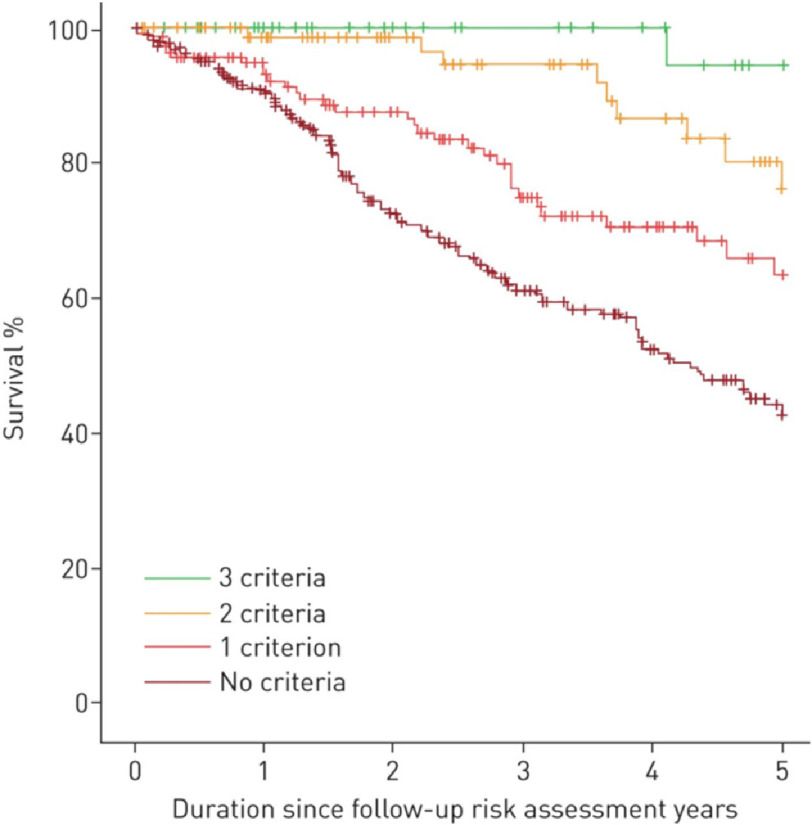
Kaplan-Meier survival estimates according to the number of low-risk criteria met at first follow-up reanalysing the COMPERA data using number of low risk criteria met (p < 0.001). Modified from [12].

Evaluation of data using the COMPERA database has suggested the number of low risk criteria that are met in terms of functional class, 6 minute walking distance and NT pro-BNP has better discriminatory functionality (Figure 3).

## Take-home points

When managing patients with PAH, risk assessment should play a vital role in the care delivered to the patient. To accurately prognosticate and provide evidence-based treatment plans to the patient should be of utmost importance. The various risk calculators, such as that from the Registry to Evaluate Early and Long-term PAH Disease Management, have been validated and are effective at providing the patient and physician with valuable information to predict mortality and prognosis and ultimately provide appropriate treatment^13^.
